# Supplementary material for: Percutaneous core-needle biopsy before and immediately after coaxial microwave ablation in solid non-small cell lung cancer: the comparison of genomic testing from specimens
Source: Cancer Imaging. 2023 Oct 3;23:93. doi: 10.1186/s40644-023-00610-6 (PMC10548670; doi:10.1186/s40644-023-00610-6)
Supplement: Supplementary file 1 — Supplementary Material 1 [file 40644_2023_610_MOESM1_ESM.docx]

**Percutaneous** **Core-needle Biopsy Before and Immediately after** **Coaxial Microwave Ablation in Solid Non-small Cell Lung Cancer: the Comparison of** **Genomic Testing from Specimens.**

Sheng Xu, MD^1*^, Lei He, MD^2*^, Jing Qi, MD^3*^, Fan-Lei Kong, MD^4^, Zhi-Xin Bie, MD^1^, Yuan-Ming Li, MD^1^, Zheng Wang, MD^2^, Xiao-Guang Li, MD^1^

1. Department of Minimally Invasive Tumor Therapies Center, Beijing Hospital, National Center of Gerontology, Institute of Geriatric Medicine, Chinese Academy of Medical Sciences, 100730, Beijing, China.

2. Department of Pathology, Beijing Hospital, National Center of Gerontology, Institute of Geriatric Medicine, Chinese Academy of Medical Sciences, 100730, Beijing, China.

3. Department of Neurology, Beijing Chaoyang Hospital, Capital Medical University, 100020, Beijing, China.

4. Department of Radiology, Qilu Hospital of Shandong University, 250063, Jinan, China.

*These authors (Sheng Xu, Lei He, and Jing Qi) contributed equally to this study.

**Corresponding author:**

Zheng Wang, MD,

Department of Pathology,

Beijing Hospital, National Center of Gerontology, Institute of Geriatric Medicine, Chinese Academy of Medical Sciences,

No.1 Da Hua Road, Dong Dan, Beijing, 100730, China;

E-mail: [wangzhengmay@163.com](mailto:wangzhengmay@163.com).

Xiao-Guang Li, MD,

Department of Minimally Invasive Tumor Therapies Center,

Beijing Hospital, National Center of Gerontology, Institute of Geriatric Medicine, Chinese Academy of Medical Sciences,

No.1 Da Hua Road, Dong Dan, Beijing, 100730, China;

Graduate School of Peking Union Medical College, Chinese Academy of Medical Sciences,

9 Dongdansantiao Street, Dongcheng District, Beijing, 100730, China;

E-mail: [xglee88@126.com](mailto:xglee88@126.com).

**Acknowledgements**

None.

**Authors’ contribution**

Sheng Xu, Zheng Wang and Xiao-Guang Li conceived of the study. Lei He participated in the design of the study and the initial syntheses of results. Jing Qi, Sheng Xu and Lei He prepared figures and wrote the manuscript text. Fan-Lei Kong, Yuan-Ming Li and Zhi-Xin Bie collected the data. Sheng Xu and Jing Qi made statistical analysis. All authors read and approved the final manuscript

**Funding**

This work was funded by the National High Level Hospital Clinical Research Funding (BJ-2020-164). Funding source had no involvements in the financial support for the conduct of the research and preparation of the article.

**Data Availability**

The datasets used and/or analyzed during the current study are available from the corresponding author upon reasonable request

**Declarations**

**Competing interests**

Not applicable.

**Ethics approval and consent to participate**

The institutional ethics review board approved this study. Informed Consent was waived due to the retrospective nature.

**Consent for publication**

Not applicable.

**Abstract**

**Purpose:** To compare the genomic testing based on specimens obtained from percutaneous core-needle biopsy (CNB) before and immediately after coaxial microwave ablation (MWA) in solid non-small cell lung cancer (NSCLC), and to investigate the diagnostic performance of CNB immediately after coaxial MWA in solid NSCLC.

**Methods:** Coaxial MWA and CNB were performed for NSCLC patients, with a power of 30 or 40 watts (W) in MWA between the pre- and post-ablation CNB, followed by continuous ablation after the second CNB on demand. The paired specimens derived from the same patient were compared for pathological diagnosis and genomic testing. DNA/RNA extracted from the paired specimens were also compared.

**Results**: A total of 33 NSCLC patients with solid lesions were included. There were two patients (6.1%) without atypical cells and three patients (9.1%) who had the technical failure of genomic testing in post-ablation CNB. The concordance rate of pathological diagnosis between the twice CNB was 93.9% (kappa=0.852), while that of genomic testing was 90.9% (kappa=0.891). For the comparisons of DNA/RNA extracted from pre- and post-ablation CNB in 30 patients, no significant difference was found when the MWA between twice CNB has a power of 30 or 40 W and ablation time within five minutes (*P*=0.174).

**Conclusions:** If the pre-ablation CNB presented with a high risk of pneumothorax or hemorrhage, the post-ablation CNB could be performed to achieve accurate pathological diagnosis and genomic testing and the maximum effect of ablation, which might allow for the diagnosis of genomic testing in 90.9% of solid NSCLC.

**Key points:**

1. CNB performed immediately after MWA might allow for the diagnosis of genomic testing in 90.9% of NSCLC;

2. The quantitative analyses of DNA/RNA extracted from specimens in post-ablation CNB were not affected when the MWA has a power of 30 or 40 W and an ablation time within five minutes.

**Keywords:** Non-small cell lung cancer; Genomic testing; Microwave ablation; Biopsy.

**Abbreviations:**

CNB Core-needle biopsy

MWA Microwave ablation

NSCLC Non-small cell lung cancer

RFA Radiofrequency ablation

SIR Society of Interventional Radiology

GGO Ground-glass opacity

ECOG Eastern Cooperation Oncology Group

PS Performance status

CT Computed tomography

AEs Adverse events

**Introduction**

Primary lung cancer remains the leading cause of cancer mortality and ranks second in cancer morbidity globally [1]. In China, non-small cell lung cancer (NSCLC) accounts for 85% of the lung cancer subtypes [2]. The prognosis of lung cancer has improved and the advance of molecular targeted therapy contributed to it [3]. Gene mutations strongly affect the formation and progression of NSCLC, and represent the potential therapeutic targets [4, 5]. Therefore, pathological diagnosis and genomic testing allow for the diagnosis of NSCLC and the detection of gene mutations [6]. Tumor samples used for pathological diagnosis and genomic testing in unresectable suspicious NSCLC are predominantly derived from percutaneous core-needle biopsy (CNB), which has the advantages of being minimally invasive, repeatable, and suitable for peripheral pulmonary lesions [6, 7].

Thermal ablation has been recommended as a treatment option for unresectable early-stage NSCLC, with the mechanism of inducing a zone of coagulative necrosis encompassing the tumor and its margin [6, 8]. Of these, microwave ablation (MWA) and radiofrequency ablation (RFA) are the primary ablative techniques. Obtaining precise pathological and genomic information is essential for NSCLC treated with thermal ablation [9]. It should be noticed that the single procedure of CNB may cause a high risk of complications (hemorrhage, air embolism, etc.), especially for lung lesions that with small diameters or adjacent to vessels, which may affect the accuracy of obtaining specimens and interfere with precise tumor positioning [10, 11]. Therefore, the standards from Society of Interventional Radiology (SIR) recommend that synchronous lung biopsy and ablation can be considered if the hemorrhage might occur during the biopsy and disturb the ablation, while the sequence of biopsy and ablation has not been mentioned [12].

In clinical practice, a high risk of occurring hemorrhage still existed in the biopsy immediately before ablation despite a synchronous procedure, especially for the tumors with hypervascularity or adjacent to vessels, which may lead to indeterminate tumor positioning and increase the risk of incomplete ablation [11]. Therefore, several studies attempted the post-ablation biopsy to achieve accurate diagnosis and treatment, and improve safety concomitantly. It is reported that the biopsy immediately after ablation enables the identification of histology subtypes and is per that of pre-ablation biopsy, with a pathological diagnosis rate of 70-100% [13-18]. Whether the accuracy of genomic testing could also be achieved in biopsy immediately after ablation remains debatable. As far as we know, only two studies have investigated it [13, 15]. Hasegawa et al. [15] found that EGFR and KRAS mutations can be analyzed in 74% of the specimens from post-ablation biopsy, but almost 50% of the patients are lung metastases. Another study showed that success rates of genomic testing were comparable between pre- and post-ablation biopsy in ground-glass opacity (GGO) nodules, and only EGFR mutation was detected [13]. If these results apply to solid NSCLC and other gene mutations remains unclear. Therefore, this study was conducted to compare the genomic testing based on specimens obtained from percutaneous CNB before and immediately after coaxial MWA in solid NSCLC, and to investigate the diagnostic performance of CNB immediately after coaxial MWA in solid NSCLC, with the innovations of more gene mutants being detected and the quantitative analyses of DNA/RNA extracted from specimens.

**Materials and Methods**

Patient criteria

All suspicious or confirmed NSCLC patients treated with coaxial MWA and pre- and post-ablation CNB between November 2021 and August 2022 at our institution were included. This single-center retrospective study was conducted per the Declaration of Helsinki. The institutional ethics review board approved this study. Inclusion criteria consist: (a) age ≥18 years; (b) solid NSCLC, with a tumor diameter ≥ 1 cm; and (c) Eastern Cooperation Oncology Group (ECOG) performance status (PS) of 0-3. Exclusion criteria are: (a) GGO nodules; (b) pathological diagnoses of small cell lung cancer or lung metastases; and (c) incomplete data.

The evaluation of positron emission tomography or contrast-enhanced computed tomography (CT) was undergone before MWA, which assisted the tumor staging via the clinical TNM staging system (eighth edition) [19]. All laboratory examinations were conducted within one week before the procedures.

Procedures of coaxial MWA and CNB

The MWA and CNB procedures followed the SIR guidelines [12, 20], and were performed by several experienced interventional radiologists under the guidance of CT (CT590; GE Healthcare, USA). The indications for MWA included early-stage NSCLC or patients who are resistant/intolerant to standard treatments (chemo-radiotherapy, surgery, or tyrosine kinase inhibitors). The goals of MWA are to achieve complete ablation in primary tumors with small diameters or to inactive the lesions as much as possible in primary tumors with large diameters. The goals of CNB are to obtain genomic testing for previously diagnosed NSCLC or to achieve both pathological diagnosis and genomic testing for suspicious NSCLC. As described previously [21], an MTC-3C MWA system (Vison Medicine, China) was used for ablation, with a microwave emission frequency of 2,450±50 MHz and an adjustable power of 20-80 W. The MWA antennas (Vison) were 15–18 cm in effective length and 16-18 G in outside diameter, with a 15 mm active tip. Preprocedural CT was performed to conduct the ablation plan and to clarify the suitable position, puncture site location, optimal puncture trajectory, and the number of MWA antennas. Local anesthesia was used for most patients, while intravenous anesthesia was used for patients requiring more pain control. During the coaxial procedures, a 15 G coaxial introducer needle (Argon Medical Devices, USA) was first introduced into the tumor, and then the stylet was replaced with a 16 G full-core biopsy needle (BioPince; Argon) through the cannula for pre-ablation CNB, followed by an MWA antenna (Vison) being advanced into the tumor and MWA was performed at a power of 30 or 40 watts (W) and planned duration, with adjustments of the antenna as needed. After the initial ablation, the MWA antenna was replaced with another 16 G full-core biopsy needle through the cannula, and post-ablation CNB was conducted at the same site with pre-ablation CNB. Then, the continuous ablation was performed after the second CNB as needed. The procedure was terminated when the ablation zone included a 5-10 mm rim of GGO beyond the tumor margins or the tumor was inactivated as much as possible by palliative ablation. Finally, a repeat CT scan was undergone to evaluate the ablation zone and detect the adverse events (AEs).

Pathological diagnosis and genomic testing

Specimens were preserved in formalin and were transferred for pathological diagnosis and genomic testing. The pathological diagnosis was evaluated after hematoxylin & eosin and immunohistochemistry staining. The DNA/RNA was extracted from formalin-fixed, paraffin-embedded specimens via an FFPE DNA Extraction Kit (Amoy Diagnostics). The genomic testing of all specimens was performed in an AmoyDx Multi-Gene Mutations Detection Kit (Amoy Diagnostics), which harbored DNA-based mutation and RNA-based fusion detection real-time PCR assays, and can detect the mutational status of EGFR, KRAS, BRAF, NRAS, HER2, PIK3CA, ALK, ROS1, MET, and RET simultaneously [22].

Follow-up and assessment

Follow-up with CT was conducted one day and one month after MWA to detect AEs, including pneumothorax, pleural effusion, pulmonary hemorrhage, etc. AEs were assessed per the National Cancer Institute Common Terminology Criteria for Adverse Events, version 5.0 [23]. For the management of AEs, antalgic, antipyretic, or hemostatic treatments were administered for patients who presented with grade-2 AEs or higher, and chest tube placement was performed for patients with moderate and severe pneumothorax, pleural effusion, or hemothorax and was terminated when these AEs disappeared. Technical failure of CNB was defined as the absence of atypical cells for pathological diagnosis or the limited amounts of specimens that cannot be used for genomic testing.

Statistical analyses

SPSS 25.0 (IBM Corp., USA) was used for statistical analyses. Demographic characteristics, AEs, pathological diagnosis, and genomic information were evaluated. The diagnostic performance was evaluated by the concordance rate of pathological diagnosis and genomic testing in pre- and post-ablation CNB, which refers to the proportion of identical results. Agreements of pathological diagnosis and genomic testing between twice CNB were evaluated using Cohen’s kappa statistics. DNA/RNA of the specimens obtained from twice CNB were also compared, with paired Student’s t test for data that obeys normal distribution and Wilcoxon matched-pairs signed rank test for data that disobeys normal distribution. A *P*-value < 0.05 was considered statistically significant.

**Results**

Demographic characteristics

A total of 33 NSCLC patients with sold lesions were enrolled (Figure 1), with a mean tumor diameter of 4.4±2.2 cm. Of these, 72.7% (24/33) of the patients were at an advanced stage. Patient characteristics are presented in Table 1. The MWA between pre- and post-ablation CNB was performed at a power of 30 W for 18 patients (54.5%) and 40 W for 15 patients (45.5%), with a mean ablation time of 7.9±5.5 minutes. The maximum power of MWA in the entire procedure was 40.0±7.9 W while the total ablation time was 11.4±5.5 minutes.

AEs

Pneumothorax and post-ablation syndrome were the two most common AEs, with both incidence rates of 12.1% (4/33). Detailed AEs are presented in Table 2. There were three patients (9.1%) with moderate or severe pneumothorax, pleural effusion, or hemothorax who required chest tube placement, and all of them recovered. Moreover, no one occurs severe AEs after the coaxial procedures.

Pathological diagnosis and genomic testing analysis

Adenocarcinoma was the predominant tumor subtype in pre- and post-ablation CNB, with a percentage of 75.8% (25/33) and 72.7% (24/33), respectively. Details of pathological diagnosis are presented in Table 3. There were two patients (6.1%) who were absent of atypical cells in post-ablation CNB. The concordance rate of pathological diagnosis between twice CNB was 93.9%, with a kappa value of 0.852 (*P*<0.001). Scale maps of the gene mutations are shown in Figure 2. Of these, EGFR mutation was the predominant gene mutation in pre-ablation CNB, with an incidence of 33.3% (12/33), followed by KRAS (15.2%), MET (12.1%), ROS1 (6.1%), and BRAF (3.0%). There were three patients (9.1%) who had a technical failure of genomic testing in post-ablation CNB. The concordance rate of genomic testing between twice CNB was 90.9%, with a kappa value of 0.891 (*P*<0.001).

Genomic testing at the DNA/RNA level using specimens from CNB

The DNA/RNA extracted from specimens between twice CNB was quantitated and presented in 30 patients (Table 3 and Figure 3). The DNA and RNA in post-ablation CNB were 20.4±25.1 and 7.2±8.6 ng/μl, respectively, which was significantly lower than that of 57.1±57.8 (*P*<0.001) and 17.3±21.8 ng/μl (*P*=0.003; Figure 4) in pre-ablation CNB, respectively. The impacts of ablation time and power between the twice CNB on DNA/RNA levels were shown in Table 4, with no significant difference being found when the MWA has a power of 30 or 40 W and ablation time within five minutes (*P*=0.174).

**Discussion**

It was reported that approximately 50-70% of Asian and 30-40% of non-Asian NSCLC patients harbor gene mutations [24]. Of these, EGFR mutation occurs in 55% of Asian patients and 15% of non-Asian patients with lung adenocarcinoma, followed by ALK rearrangement occurs in 5-8%, and other mutations are limited to 5% of non-squamous NSCLC [25, 26]. According to the latest cancer statistics in the USA, the median overall survival of lung cancer increased to 13 months and the three-year relative survival rate was up to 38% [3]. Molecular targeted therapy prompts this progression, which is mainly against gene mutations and is recommended as the standard treatment [6]. Percutaneous CNB was one of the primary methods to obtain specimens for pathological diagnosis and genomic testing, and was especially suitable for peripheral or unresectable lung lesions, with a diagnostic accuracy rate of 90% [6, 20, 27]. It should be noticed that the patients were at a high risk of occurring pulmonary hemorrhage or pneumothorax when CNB was performed for pulmonary lesions that with hypervascularity or were adjacent to vessels or bronchi, which may influence the precise biopsy.

Thermal ablation was recommended as a treatment option for stage I NSCLC patients who have contraindications to surgery or radiotherapy, or be considered as a salvage treatment for patients who developed progression on EGFR or ALK therapy [6]. Thermal ablation can not only conduct the coagulative necrosis of tumor tissues but also cause the collapse of small or medium-sized blood vessels depending on the hyperthermia directly, to some extent, has hemostatic effects [28]. In general, the biopsy was supposed to be performed before ablation to obtain accurate diagnoses. Nevertheless, a high risk of occurring hemorrhage existed in pre-ablation CNB, especially for GGO nodules and lesions with small diameters or adjacent to vessels, which may disturb the subsequent ablation [11]. In 2012, a retrospective study analyzed 33 lung neoplasm patients treated with simultaneous CNB and RFA, and found a local tumor control rate of 77% in a median follow-up of one year [29]. Then, Wang et al. [30] attempted simultaneously coaxial MWA and biopsy in suspicious malignant lung lesions and found this procedure has lesser AEs but similar efficacy when compared with separate procedures, which could achieve the diagnosis and treatment concomitantly and was recommended by SIR standards for the lesions with a high risk of hemorrhage that may interfere with the ablation [12]. Nevertheless, a high risk of occurring hemorrhage still existed in the biopsy immediately before ablation despite a synchronous procedure, which may lead to indeterminate tumor positioning and increase the risk of incomplete ablation [11]. Therefore, several authors attempted to perform CNB immediately after ablation in highly suspicious malignant lung lesions, and indicate the accuracy and safety of this procedure [13-18].

It was reported that the pathological diagnosis rate of CNB immediately after thermal ablation ranged from 70% to 100% [13-18], with the potential mechanisms of apoptosis progressing in tumor cells subjected to hyperthermia gradually, and cell morphology remaining in the tumor within the first month after ablation [14, 31-33]. In 2016, Hasegawa et al. [14] performed a biopsy immediately after RFA for three patients with lung malignancy, including two metastases and one adenocarcinoma, all of whom achieved the precise pathological diagnosis. Then, a study attempted coaxial biopsy immediately after RFA, and found histological subtype can be distinguished in 70% of patients despite most of the tumors being lung metastases [16]. Wei et al. [18] performed CNB immediately after MWA in 69 confirmed NSCLC patients, and found the pathological diagnosis can be distinguished in 85.3% of patients and 69.1% of patients have identical histological subtypes when compared with previous results, which indicated that the accuracy of post-ablation CNB for determining the tumor subtypes. Another study conducted by Hasegawa et al. [15] enrolled 13 solid pulmonary lesions and six GGO nodules that had undergone CNB immediately after RFA, with the overall pathological diagnosis rate reaching 79% while that was only 50% for GGO nodules. In a study of 74 patients with GGO nodules, the pathological diagnosis rates of pre- and post-MWA CNB were 85.1% and 74.3%, respectively, and the histological subtypes could also be distinguished, which indicated the comparability of pre- and post-ablation CNB [17]. Compared to the above studies, all of the patients enrolled in our study were NSCLC with solid lesions, and pathological diagnoses between pre- and post-ablation CNB were compared directly, with a high concordance rate of 93.9%. Two patients (6.1%) presented with the absence of atypical cells in post-ablation CNB and the potential interpretation was the overlong ablation time between twice CNB that lead to the carbonization of specimens. Moreover, the attenuation of immunohistochemistry staining in post-ablation CNB was also found, which is per the results from a previous study [15].

Two studies have investigated the accuracy of genomic testing in CNB immediately after thermal ablation, with the technical success rate ranging from 74% to 84% [13, 15]. In 2018, a study reported that EGFR and KRAS mutations can be detected in 74% of the specimens obtained from post-ablation CNB although GGO lesions were included and the percentage of NSCLC was less than 50% [15]. Then, Chi et al. [13] presented a success rate of 100% and 84% could be achieved for pre- and post-MWA CNB in GGO nodules, respectively, with no significant difference being found despite only EGFR mutation being detected. Of these, the MWA between twice CNB was at a power of 20 W and this procedure could decrease the incidence of AEs. However, the quantitative analysis of DNA/RNA extracted from specimens is scarce previously. Our study verified the applicability of these results in solid NSCLC and found a concordance rate of 90.9% between pre- and post-ablation CNB, which was higher than that in previous studies [13, 15]. Besides, the scope of gene mutations was extended, with the verification of accuracy in MET, ROS1, and BRAF. Adequate amounts of DNA/RNA extracted from specimens were critical for the quantitative analyses of genomic testing. In this study, the mean concentration of DNA/RNA in post-ablation CNB was significantly lower than that in pre-ablation CNB, which demonstrated that the post-ablation biopsy influences the DNA/RNA level, in other words, has an impact on quantitative analyses for genomic testing. The potential mechanism was that the heat delivered from the ablation antenna damaged the DNA in tumor cells and induced apoptosis, and the RNA structure was unstable and was also prone to be damaged by hyperthermia [34]. In theory, the more heat absorbed by the tumor cells, the more severe damage is brought to DNA/RNA. Therefore, we attempted to investigate the potential cut-off values of ablation-related parameters in MWA between twice CNB, which could not only achieve the qualitative analysis of genomic testing but also had no significant impacts on DNA/RNA levels. This study indicated that the quantitative analyses of DNA/RNA may not be influenced significantly when the MWA between pre- and post-ablation CNB was performed at a power of 30 or 40 W and ablation time within five minutes.

Several limitations in this study should be presented. First, the selection bias may exist due to the retrospective nature of this study. Second, the patients are from single-center and the sample size was still limited. Third, although both DNA- and RNA-related gene mutations were detected, the results are still needed to be verified beyond the scope of gene mutations in this study. Finally, the precise evaluation of heat distribution in the tumor was complicated and vulnerable to being affected by multiple factors, including the tumor volume, margins, density, intratumoral vascularity, blood supply, ablation power, duration, and so on, and further investigation was warranted to assess the potential impacts of these factors on genomic testing precisely.

**Conclusion**

If the pre-ablation CNB presented with a high risk of pneumothorax or hemorrhage, the post-ablation CNB could be performed to achieve accurate pathological diagnosis and genomic testing and the maximum effect of ablation, which might allow for the diagnosis of genomic testing in 90.9% of solid NSCLC.

**References:**

1. Sung H, Ferlay J, Siegel RL, Laversanne M, Soerjomataram I, Jemal A, et al. Global Cancer Statistics 2020: GLOBOCAN Estimates of Incidence and Mortality Worldwide for 36 Cancers in 185 Countries. Ca: A Cancer Journal for Clinicians. 2021;71(3):209-49. https://doi.org/10.3322/caac.21660.

2. Zheng R, Zhang S, Zeng H, Wang S, Sun K, Chen R, et al. Cancer incidence and mortality in China, 2016. Journal of the National Cancer Center. 2022;2(1):1-9. https://doi.org/10.1016/j.jncc.2022.02.002.

3. Siegel RL, Miller KD, Fuchs HE, Jemal A. Cancer statistics, 2022. Ca-Cancer J Clin. 2022;72(1):7-33. https://doi.org/10.3322/caac.21708.

4. Novello S, Barlesi F, Califano R, Cufer T, Ekman S, Levra MG, et al. Metastatic non-small-cell lung cancer: ESMO Clinical Practice Guidelines for diagnosis, treatment and follow-up. Ann Oncol. 2016;27(suppl 5):v1-27. https://doi.org/10.1093/annonc/mdw326.

5. Hanna N, Johnson D, Temin S, Baker SJ, Brahmer J, Ellis PM, et al. Systemic Therapy for Stage IV Non-Small-Cell Lung Cancer: American Society of Clinical Oncology Clinical Practice Guideline Update. J Clin Oncol. 2017;35(30):3484-515. https://doi.org/10.1200/JCO.2017.74.6065.

6. Non-Small Cell Lung Cancer. Clinical Practice Guidelines in Oncology. NSCLC 2022 V5.

7. Fintelmann FJ, Martin NA, Tahir I, Quinn EM, Allen TC, Joseph L, et al. Optimizing molecular testing of lung cancer needle biopsy specimens: potential solutions from an interdisciplinary qualitative study. Resp Res. 2023;24(1):17. https://doi.org/10.1186/s12931-023-02321-9.

8. Wei Z, Yang X, Ye X, Feng Q, Xu Y, Zhang L, et al. Microwave ablation plus chemotherapy versus chemotherapy in advanced non-small cell lung cancer: a multicenter, randomized, controlled, phase III clinical trial. Eur Radiol. 2020;30(5):2692-702. https://doi.org/10.1007/s00330-019-06613-x.

9. Kerr KM, Bubendorf L, Edelman MJ, Marchetti A, Mok T, Novello S, et al. Second ESMO consensus conference on lung cancer: pathology and molecular biomarkers for non-small-cell lung cancer. Ann Oncol. 2014;25(9):1681-90. https://doi.org/10.1093/annonc/mdu145.

10. Yun S, Kang H, Park S, Kim BS, Park JG, Jung MJ. Diagnostic accuracy and complications of CT-guided core needle lung biopsy of solid and part-solid lesions. Brit J Radiol. 2018;91(1088):20170946. https://doi.org/10.1259/bjr.20170946.

11. Kong F, Wang C, Li Y, Li X. Advances in study of the sequence of lung tumor biopsy and thermal ablation. Thorac Cancer. 2021;12(3):279-86. https://doi.org/10.1111/1759-7714.13795.

12. Genshaft SJ, Suh RD, Abtin F, Baerlocher MO, Dariushnia SR, Devane AM, et al. Society of Interventional Radiology Quality Improvement Standards on Percutaneous Ablation of Non-Small Cell Lung Cancer and Metastatic Disease to the Lungs. J Vasc Interv Radiol. 2021;32(8):1241-2. https://doi.org/10.1016/j.jvir.2021.04.027.

13. Chi J, Ding M, Wang Z, Hu H, Shi Y, Cui D, et al. Pathologic Diagnosis and Genetic Analysis of Sequential Biopsy Following Coaxial Low-Power Microwave Thermal Coagulation For Pulmonary Ground-Glass Opacity Nodules. Cardiovasc Inter Rad. 2021;44(8):1204-13. https://doi.org/10.1007/s00270-021-02782-9.

14. Hasegawa T, Kondo C, Sato Y, Inaba Y, Yamaura H, Kato M, et al. Diagnostic Ability of Percutaneous Needle Biopsy Immediately After Radiofrequency Ablation for Malignant Lung Tumors: An Initial Experience. Cardiovasc Inter Rad. 2016;39(8):1187-92. https://doi.org/10.1007/s00270-016-1324-3.

15. Hasegawa T, Kondo C, Sato Y, Inaba Y, Yamaura H, Kato M, et al. Pathologic Diagnosis and Genetic Analysis of a Lung Tumor Needle Biopsy Specimen Obtained Immediately After Radiofrequency Ablation. Cardiovasc Inter Rad. 2018;41(4):594-602. https://doi.org/10.1007/s00270-017-1845-4.

16. Tselikas L, de Baere T, Deschamps F, Hakime A, Besse B, Teriitehau C, et al. Diagnostic yield of a biopsy performed immediately after lung radiofrequency ablation. Eur Radiol. 2017;27(3):1211-7. https://doi.org/10.1007/s00330-016-4447-7.

17. Wang J, Ni Y, Yang X, Huang G, Wei Z, Li W, et al. Diagnostic ability of percutaneous core biopsy immediately after microwave ablation for lung ground-glass opacity. J Cancer Res Ther. 2019;15(4):755-9. https://doi.org/10.4103/jcrt.JCRT_399_19.

18. Wei Z, Wang Q, Ye X, Yang X, Huang G, Li W, et al. Microwave ablation followed by immediate biopsy in the treatment of non-small cell lung cancer. Int J Hyperther. 2018;35(1):262-8. https://doi.org/10.1080/02656736.2018.1494856.

19. Goldstraw P, Chansky K, Crowley J, Rami-Porta R, Asamura H, Eberhardt WE, et al. The IASLC Lung Cancer Staging Project: Proposals for Revision of the TNM Stage Groupings in the Forthcoming (Eighth) Edition of the TNM Classification for Lung Cancer. J Thorac Oncol. 2016;11(1):39-51. https://doi.org/10.1016/j.jtho.2015.09.009.

20. Sheth RA, Baerlocher MO, Connolly BL, Dariushnia SR, Shyn PB, Vatsky S, et al. Society of Interventional Radiology Quality Improvement Standards on Percutaneous Needle Biopsy in Adult and Pediatric Patients. J Vasc Interv Radiol. 2020;31(11):1840-8. https://doi.org/10.1016/j.jvir.2020.07.012.

21. Xu S, Bie ZX, Li YM, Li B, Peng JZ, Kong FL, et al. Computed tomography-guided microwave ablation for non-small cell lung cancer patients on antithrombotic therapy: a retrospective cohort study. Quant Imag Med Surg. 2022;12(6):3251-63. https://doi.org/10.21037/qims-21-1043.

22. Kong F, Li Y, Guo R, Yang L, Di J, He L, et al. Liquid biopsy assay for pulmonary adenocarcinoma using supernatants from core-needle biopsy specimens. Thorac Cancer. 2022;13(12):1822-6. https://doi.org/10.1111/1759-7714.14461.

23. Common Terminology Criteria for Adverse Events (CTCAE) v5.0. Available at: https://ctep.cancer.gov/protocolDevelopment/electronic_applications/docs/CTCAE_v5_Quick_Reference_5x7.pdf. Accessed June 7, 2023.

24. Herbst RS, Morgensztern D, Boshoff C. The biology and management of non-small cell lung cancer. Nature. 2018;553(7689):446-54. https://doi.org/10.1038/nature25183.

25. Rosas G, Ruiz R, Araujo JM, Pinto JA, Mas L. ALK rearrangements: Biology, detection and opportunities of therapy in non-small cell lung cancer. Crit Rev Oncol Hemat. 2019;136:48-55. https://doi.org/10.1016/j.critrevonc.2019.02.006.

26. Kohno T, Nakaoku T, Tsuta K, Tsuchihara K, Matsumoto S, Yoh K, et al. Beyond ALK-RET, ROS1 and other oncogene fusions in lung cancer. Transl Lung Cancer R. 2015;4(2):156-64. https://doi.org/10.3978/j.issn.2218-6751.2014.11.11.

27. Kothary N, Bartos JA, Hwang GL, Dua R, Kuo WT, Hofmann LV. Computed tomography-guided percutaneous needle biopsy of indeterminate pulmonary pathology: efficacy of obtaining a diagnostic sample in immunocompetent and immunocompromised patients. Clin Lung Cancer. 2010;11(4):251-6. https://doi.org/10.3816/CLC.2010.n.032.

28. Moll X, Fondevila D, Garcia-Arnas F, Burdio F, Trujillo M, Irastorza RM, et al. Comparison of two radiofrequency-based hemostatic devices: saline-linked bipolar vs. cooled-electrode monopolar. Int J Hyperther. 2022;39(1):1397-407. https://doi.org/10.1080/02656736.2022.2140840.

29. Schneider T, Puderbach M, Kunz J, Bischof A, Giesel FL, Dienemann H, et al. Simultaneous computed tomography-guided biopsy and radiofrequency ablation of solitary pulmonary malignancy in high-risk patients. Respiration. 2012;84(6):501-8. https://doi.org/10.1159/000342874.

30. Wang D, Li B, Bie Z, Li Y, Li X. Synchronous core-needle biopsy and microwave ablation for highly suspicious malignant pulmonary nodule via a coaxial cannula. J Cancer Res Ther. 2019;15(7):1484-9. https://doi.org/10.4103/jcrt.JCRT_721_18.

31. Yasui K, Kanazawa S, Sano Y, Fujiwara T, Kagawa S, Mimura H, et al. Thoracic tumors treated with CT-guided radiofrequency ablation: initial experience. Radiology. 2004;231(3):850-7. https://doi.org/10.1148/radiol.2313030347.

32. Wang Q, Huang J, Ma K, Li T, Chen M, Wang S, et al. Evaluation of ghost cell survival in the area of radiofrequency ablation. Plos One. 2012;7(12):e53158. https://doi.org/10.1371/journal.pone.0053158.

33. Clasen S, Krober SM, Kosan B, Aebert H, Fend F, Bomches A, et al. Pathomorphologic evaluation of pulmonary radiofrequency ablation: proof of cell death is characterized by DNA fragmentation and apoptotic bodies. Cancer-Am Cancer Soc. 2008;113(11):3121-9. https://doi.org/10.1002/cncr.23882.

34. Mantso T, Goussetis G, Franco R, Botaitis S, Pappa A, Panayiotidis M. Effects of hyperthermia as a mitigation strategy in DNA damage-based cancer therapies. Semin Cancer Biol. 2016;37-38:96-105. https://doi.org/10.1016/j.semcancer.2016.03.004.

**Table 1. Clinical characteristics of NSCLC patients treated with coaxial MWA and pre- and post-ablation CNB.**

| Variables | NSCLC patients(n=33) | Variables | NSCLC patients(n=33) |
| --- | --- | --- | --- |
| Age(y) | 68.4±12.0 | Radiological features |  |
| Gender |  | Tumor diameter (cm) | 4.4±2.2 |
| Male | 19(57.6%) | Location |  |
| Female | 14(42.4%) | Lower or middle lobe | 16(48.5%) |
| Comorbidity |  | Upper lobe | 17(51.5%) |
| Hypertension | 16(48.5%) | Emphysema | 12(36.4%) |
| CCVd | 12(36.4%) | Malignant pleural effusion | 4(12.1%) |
| DM | 5(15.2%) | Extrapulmonary metastases | 10(30.3%) |
| Tumor stage |  | Laboratory examinations |  |
| I | 6(18.2%) | WBC(*10^9^/L) | 6.5±1.9 |
| II | 3(9.1%) | Hb(g/L) | 128.4±18.6 |
| III | 7(21.2%) | PLT(*10^9^/L) | 230.4±68.2 |
| IV | 17(51.5%) | PT(s) | 11.5±1.2 |
| ECOG score |  | CEA(ng/ml) | 18.9±42.5 |
| 0 | 12(36.4%) | MWA-related factors |  |
| 1 | 13(39.4%) | Ablation time between twice CNB (min) | 7.9±5.5 |
| 2 | 5(15.2%) | Power between twice CNB (W) |  |
| 3 | 3(9.1%) | 30 | 18(54.5%) |
| Treatment history |  | 40 | 15(45.5%) |
| Surgery | 4(12.1%) | Maximum power (W) | 40.0±7.9 |
| TKIs | 5(15.2%) | Total ablation time (min) | 11.4±5.5 |

Note. Frequencies and percentages are reported for categorical variables, and the mean ± standard deviation is reported for continuous variables. NSCLC=Non-small cell lung cancer. CNB=Core-needle biopsy. MWA=Microwave ablation. CCVd=Cardiocerebrovascular diseases. DM=Diabetes mellitus. ECOG=Eastern Cooperation Oncology Group. TKIs=Tyrosine kinase inhibitors. WBC=White blood cell. PLT=Platelet. PT=Prothrombin time. Hb=Hemoglobin. CEA=Carcinoembryonic antigen.

**Table 2. Details of AEs in NSCLC treated with coaxial MWA and pre- and post-ablation CNB.**

| Variables | NSCLC patients(n=33) |
| --- | --- |
| Grade 1 AEs |  |
| Pneumothorax | 3(9.1%) |
| Pneumonia | 1(3.0%) |
| Pleural effusion | 1(3.0%) |
| Post-ablation syndrome | 4(12.1%) |
| Pulmonary hemorrhage | 2(6.1%) |
| Grade 2 AEs |  |
| Pneumothorax | 1(3.0%) |
| Pleural effusion | 2(6.1%) |
| Hemothorax | 1(3.0%) |

AEs=Adverse events. NSCLC=Non-small cell lung cancer.

MWA=Microwave ablation. CNB=Core-needle biopsy.

**Table 3. Pathological diagnosis and DNA/RNA extracted from the specimens in pre- and post-ablation CNB.**

| Variables | Pre-ablation CNB | Post-ablation CNB | *P*-value |
| --- | --- | --- | --- |
| Tumor subtypes |  |  |  |
| Adenocarcinoma | 25(75.8%） | 24(72.7%） |  |
| Squamous cell carcinoma | 7(21.2%) | 6(18.2%) |  |
| Large cell neuroendocrine carcinoma | 1(3.0%) | 1(3.0%) |  |
| No atypical cells | 0 | 2(6.1%) |  |
| DNA (ng/μl) | 57.1±57.8 | 20.4±25.1 | ＜0.001 |
| RNA (ng/μl) | 17.3±21.8 | 7.2±8.6 | 0.003 |

NSCLC=Non-small cell lung cancer. CNB=Core-needle biopsy. DNA=Deoxyribonucleic acid. RNA=Ribonucleic acid.

**Table 4. The impacts of ablation time and power between the twice CNB on DNA/RNA in 30 NSCLC patients.**

| Variables | DNA | | *P-value* | RNA | | *P-value* |
| --- | --- | --- | --- | --- | --- | --- |
|  | Pre-ablation CNB | Post-ablation CNB |  | Pre-ablation CNB | Post-ablation CNB |  |
| Power |  |  |  |  |  |  |
| 30 W | 65.4±61.9 | 17.7±18.6 | 0.016 | 22.3±29.4 | 7.8±10.7 | 0.025 |
| 40 W | 47.5±53.4 | 16.8±25.7 | 0.007 | 18.3±20.0 | 6.5±5.8 | 0.049 |
| Ablation time |  |  |  |  |  |  |
| ＜5min | 72.4±87.4 | 16.6±13.0 | 0.168 | 16.0±15.2 | 5.9±3.5 | 0.174 |
| ≥5min | 53.3±49.8 | 21.4±27.5 | ＜0.001 | 17.6±23.4 | 7.5±9.5 | 0.006 |

CNB=Core-needle biopsy. DNA=Deoxyribonucleic acid. RNA=Ribonucleic acid.

**Figure Captions**

**Figure 1. Patient selection flowchart.**

**Figure 2. Scale maps of the gene mutations in NSCLC patients.**

**Figure 3. Quantitative analyses of DNA/RNA extracted from specimens in pre- and post-ablation CNB.**

**Figure 4. A typical case of NSCLC treated with coaxial MWA and pre- and post-ablation CNB.** a. A NSCLC patient has a new solid lesion (white arrow) and moderate pleural effusion (black arrow) in the left lung, with a treatment history of bilateral segmentectomy and the pathological diagnosis of adenocarcinoma. b. CT-guided coaxial MWA and CNB were undergone, with the pre-ablation CNB being performed for the pathological diagnosis and genomic testing. c. MWA was performed, with 30W of energy released and seven minutes of ablation time. d. Post-ablation CNB was undergone in the same site as the pre-ablation CNB, and the specimens were also used for pathological diagnosis and genomic testing. e. The chest tube was inserted to drain the pleural effusion (black arrow), with the finding of adenocarcinoma cells in pleural effusion. f. The 24 h CT reexamination reveals the ablation zone and the reduction of malignant pleural effusion. g. The specimens obtained from pre-ablation CNB confirmed the diagnosis of adenocarcinoma and the gene mutation of BRAF V600E. The H&E stain showed that the tumor was adenoidal, with a clear adenoidal structure, round and ovoid nuclei, uniform chromatin, clear cell boundaries, and interstitial fibrosis. The DNA and RNA extracted from specimens were 84.9 and 19.3 ng/μl, respectively. h. The specimens obtained from post-ablation CNB confirmed the diagnosis of adenocarcinoma and the gene mutation of BRAF V600E. The H&E stain showed that the tumor was adenoidal with deformation, sharp margins, nuclei with elevated flow-like changes, deep chromatin staining, unclear cell boundaries, and obvious signs of interstitial cauterization. The DNA and RNA extracted from specimens were 20.0 and 4.5 ng/μl, respectively, with a significant decrease from that of pre-ablation CNB.
