# Supplementary material for: Percutaneous core-needle biopsy before and immediately after coaxial microwave ablation in solid non-small cell lung cancer: the comparison of genomic testing from specimens
Source: Cancer Imaging. 2023 Oct 3;23:93. doi: 10.1186/s40644-023-00610-6 (PMC10548670; doi:10.1186/s40644-023-00610-6)
Supplement: Supplementary file 2 — Supplementary Material 2 [file 40644_2023_610_MOESM2_ESM.docx]

August. 14, 2023

Dear Prof. Stefan Diederich, Deputy Editor, Cancer Imaging.

Thank you very much for your kind reminder on the manuscript ***CAIG-D-23-00281***, entitled *“Percutaneous Core-needle Biopsy Before and Immediately after Coaxial Microwave Ablation in Solid Non-small Cell Lung Cancer: the Comparison of Genomic Testing from Specimens*”.

We have made revisions based on the requirements from the reviewers. All the revisions have been corrected in the revised manuscript. In addition, we have attached another annotated version to facilitate your review. We believe that the revised manuscript has been greatly improved. If there are still some issues, please do not hesitate to contact us. We are looking forward to hearing your favorable decision.

Sincerely Yours,

**Corresponding author:**

Sincerely Yours,

Xiao-Guang Li, MD,

Department of Minimally Invasive Tumor Therapies Center,

Beijing Hospital, National Center of Gerontology, Institute of Geriatric Medicine, Chinese Academy of Medical Sciences,

No.1 Da Hua Road, DongDan, Beijing, 100730, China;

Graduate School of Peking Union Medical College, Chinese Academy of Medical Sciences,

9 Dongdansantiao Street, Dongcheng District, Beijing, 100730, China；

E-mail: [xglee88@126.com](mailto:xglee88@126.com).
